# Mobility and increased risk of HIV acquisition in South Africa: a mixed-method systematic review protocol

**DOI:** 10.1186/s13643-018-0703-z

**Published:** 2018-02-27

**Authors:** Armstrong Dzomba, Kaymarlin Govender, Tivani P. Mashamba-Thompson, Frank Tanser

**Affiliations:** 1 Africa Health Research Institute (AHRI), K-RITH Tower Building, 719 Umbilo Road, Durban Private Bag X7, Congella, Durban, South Africa; 2 Health Economics and HIV/AIDS Research Division, Durban, South Africa; 3 Howard College, University of KwaZulu-Natal, King George V Avenue, Durban, 4001 South Africa; 4 Discipline of Public Health Medicine, School of Nursing and Public Health, University of KwaZulu-Natal, Durban, South Africa; 5 Africa Health Research Institute, (AHRI), School of Nursing and Public Health, Durban, South Africa; 6 Centre for the AIDS Programme of Research in South Africa—CAPRISA, Congella, Durban, South Africa; 7 Discipline of Public Health Medicine, Africa Health Research Institute (AHRI) and University of KwaZulu-Natal, K-RITH Tower Building, 719 Umbilo Road, Durban Private Bag X7, Congella, Durban, South Africa

**Keywords:** Sexual risk behavior, Migrants, HIV risk acquisition, South Africa

## Abstract

**Background:**

In South Africa (home of the largest HIV epidemic globally), there are high levels of mobility. While studies produced in the recent past provide useful perspectives to the mobility-HIV risk linkage, systematic analyses are needed for in-depth understanding of the complex dynamics between mobility and HIV risk. We plan to undertake an evidence-based review of existing literature connecting mobility and increased risky sexual behavior as well as risk of HIV acquisition in South Africa.

**Methods/design:**

We will conduct a mixed-method systematic review of peer-reviewed studies published between 2000 and 2015. In particular, we will search for relevant South African studies from the following databases: MEDLINE, EMBASE, Web of Science, and J-STOR databases. Studies explicitly examining HIV and labor migration will be eligible for inclusion, while non-empirical work and other studies on key vulnerable populations such as commercial sex workers (CSW) and men who have sex with men (MSM) will be excluded.

**Discussion:**

The proposed mixed-method systematic review will employ a three-phase sequential approach [i.e., (i) identifying relevant studies through data extraction (validated by use of Distiller-SR data management software), (ii) qualitative synthesis, and (iii) quantitative synthesis including meta-analysis data]. Recurrent ideas and conclusions from syntheses will be compiled into key themes and further processed into categories and sub-themes constituting the primary and secondary outcomes of this study. Synthesis of main findings from different studies examining the subject issue here may uncover important research gaps in this literature, laying a strong foundation for research and development of sustainable localized migrant-specific HIV prevention strategies in South Africa.

**Systematic review registration:**

Our protocol was registered with PROSPERO under registration number: CRD 42017055580. (https://www.crd.york.ac.uk/PROSPERO/display_record.asp?ID=CRD42017055580)

**Electronic supplementary material:**

The online version of this article (10.1186/s13643-018-0703-z) contains supplementary material, which is available to authorized users.

## Background

The vulnerability of labor migrants to HIV is a critical public health concern globally [1–3]. Persons living between their household of origin and the destination region epidemiologically carry an increased risk, vulnerability, and burden with respect to HIV infection—due to a combination of biological, socioeconomic, and structural factors [4]. Yet, with the massive scale of the generalized HIV epidemic in South Africa, transmission remains concentrated in certain geographical regions and in subpopulations known to practice high-risk sexual behaviors [5]. For example, rural communities increasingly constitute socio-geographic settings for HIV outcomes.

Evidence linking migration and HIV often conflicts and largely remains inconclusive. For instance, literature from public health and social epidemiology has long held that the association between migration and HIV transmission primarily lies in the nature of the migration process [6, 7]. The presumption here is that mobility almost invariably involves the positive selection of prime-aged men (i.e., who are either married or not married, often migrate without their wives and partners), leaving their rural communities for work [8–12]. Under such arrangements, migration has primarily been implicated as one of the key drivers of the HIV epidemic in South Africa. Yet the rise of female labor migration associated with the peak of the HIV epidemic in South Africa [13, 14] seems to challenge this male-infector model [5]. Authors note that after the year 2000, women increasingly enlisting for informal sector work in urban settings, often facing limited access to workplace health services and prevention programs, experienced high-risk exposure [13]. In fact, those migrating to metropoles such as Durban and Johannesburg or Pretoria increased their risk of acquiring HIV to as much as 75%, driving the epidemic [15]. Other literature note that migration generally produces risk through temporal disengagement of individuals from traditional sexual networks and compensation thereof by establishing newer sexual networks in the urban destination where there is a greater occurrence of risky sex linked to HIV transmission [16–18]. However, a fuller description of these risky sexual practices and characterization of odds for new HIV diagnoses associated with migration in South Africa has not been detailed elsewhere.

Notably, an illustration of multilevel determinants (i.e., policy and socio-cultural context) that link labor migration and HIV risk is presented in Weine and Kashuba [1]. In their systematic review however, sexual practice determinants by geographical region featured results from only seven South African studies of the 97 included and no meta-analysis was performed. To elicit more South Africa studies and gain a deeper understanding of the sexual practice determinants linked to migration and HIV relationship, we undertake to use a novel method of conducting systematic reviews and meta-analyses of peer-reviewed studies. Specifically, we employ a mixed-method approach [19, 20] to systematically review (describe) the existing evidence literature linking mobility and HIV [i.e., (1) HIV risk behaviors and (2) risk of HIV acquisition] in South Africa and conduct a meta-analysis, (quantifying the relationship).

## Materials and methods

This mixed-method systematic review title is published in the international prospective register for systematic reviews, PROSPERO, under the following entry number: CRD42017055580.

Title registration is accessible via this link below: https://www.crd.york.ac.uk/PROSPERO/display_record.asp?ID=CRD42017055580

### Summary of mixed-method review

The proposed mixed-method systematic review will employ a sequential approach to structure the three constituent phases of this study [21]. Phase one will be an adaptation of Arksey and O′ Malley’s [22] framework for conducting systematic reviews [i.e., involving (i) target bodies of evidence, (ii) study selection, and (iii) data extraction and appraisal. Phases two and three will comprise collating, summarizing, and reporting findings from primary studies, discussed under topics (iv) data synthesis and (v) assessment of publication bias. Primarily, phase two will be devoted to thematic analysis of the qualitative component in qualitative, quantitative, and mixed-method studies; this amounts to qualitative synthesis. This will involve a rigorous process in which ideas and conclusions proceeding from the literature are categorized into key themes (i.e., either in text format and descriptive statistics) and further synthesized into sub-themes relevant to the primary outcomes of the prospective review (i.e., HIV risk and HIV infection in migrants). This important description of emerging themes although altogether a standalone phase may be useful in framing and elaborating the subsequent quantitative synthesis. In phase three, quantitative (data) synthesis may include studies considered in phase two although studies purely quantitative and those reporting effect sizes for the mobility-HIV risk relationship will be prioritized. A meta-analysis based on studies powered for bivariate and multivariate analyses of HIV risk acquisition of migrants will be performed. Notably, our reliance on this sequential two-phase synthesis may allow us to use qualitative findings to explain the pooled quantitative results.

#### Phase 1

The primary question here is the following: How does empirical evidence linking mobility and HIV in South Africa characterize sexual risk behavior and estimate HIV infection?

The research sub-questions are:

1. What is the nature and extent of sexual practices linked to HIV among migrants in South Africa?
2. What is the risk of HIV acquisition among migrants in South Africa?

(i) Target bodies of evidence

The study will rely on the search criteria SPIDER [22], which stands for Sample, Phenomenon of interest, Design, Evaluation and Research Type. SPIDER is a qualitative/mixed-method studies framework specifically defining key elements of the proposed review question as detailed below.

##### Sample

The study will specifically include men and women migrating in South Africa.

##### Phenomenon of interest

The research included will focus on the involvement of individuals in migration.

##### Defining migration

We will define migration as a process generally involving the temporary movement of adult men and women from their normal places of residence to destination regions within South Africa. The most common stream of migration in South Africa is rural to urban migration, a system uniquely rooted in Apartheid era policies aimed at ensuring the supply of rural African male laborers in urban industrial centers such as Johannesburg.

##### Design

The study designs included will be interviews, focus groups, ethnographic observations, phenomenological, participatory action research, case series, and surveys.

##### Evaluation

The outcomes to be evaluated in the review are twofold: First, to explore attitudes, beliefs, and risky sexual behavior that increase chances for HIV infection, such as low risk perception, having multiple sexual partners, soliciting sex workers, and unprotected sex. Secondly, HIV infection in migrants confirmed through a positive HIV test result.

##### Research type

Empirical research, i.e., qualitative and mixed-method studies set in South Africa and published between 2000 and 2015 will be included.

Additionally, we will use an alternative search strategy PEOM [23], which stands for Population, Exposure, Outcomes and Measurement properties to identify epidemiological studies in particular. PEOM importantly guides the assessment of studies addressing the topic of interest, for instance by specifying in each case the type of evidence targeted.

##### Population

The study population are individuals leaving their households temporarily in South Africa.

##### Exposure

The study is interested in migration defined as a temporary movement of persons involving a change of residence from a usual place of abode.

##### Outcomes

The study will examine attitudes, beliefs, and risky behavior associated with HIV infection in migrants. Most importantly, our main outcome of interest is risk of HIV acquisition verifiable through a positive HIV test associated with being a migrant.

##### Measurement

Descriptive data and multivariate statistics quantifying the outcomes of interest, such as percentages, proportions, and measures of central tendency including odds ratios and adjusted odds ratios from observational studies such as cross-sectional and cohort designs will be considered.

##### Eligibility criteria for considering studies for the review

The inclusion/exclusion criteria developed for this review aims to accurately identify and select relevant primary studies in accordance to the descriptions of target bodies of evidence above.

- Inclusion criteria

*Study designs and participants*: focus group, ethnographic, phenomenological, participatory action research, and observational studies (cross-sectional and cohort designs) directly examining labor migration and HIV will be eligible for inclusion. However, selection will depend on whether exposure to labor migration was treated as the phenomenon of interest and/or that a labor migrant population was sampled.

*Outcomes*: Attitudes, beliefs, and HIV risk behaviors such as low HIV risk perception and multiple sexual partnering and risk of HIV acquisition.

*Study setting*: Empirical studies conducted in rural or urban settings within South Africa.

*Time period*: Studies published between January 1, 2000, and December 31, 2015, in databases consulted.

*Language*: Only studies published in English for reasons of practicality.

- Exclusion criteria

Studies on other key populations such as commercial sex workers (CSW) and men who have sex with men (MSM) will be excluded (reasons for this are given in the “Discussion” section below).

Non-empirical studies (e.g., discursive papers, theoretical, and opinion pieces) for qualitative studies and in observational studies and those lacking effect sizes such as odds ratios and/or adjusted odds ratios quantifying the increase in risk of HIV acquisition due to migration will be excluded.

Literature sourced from four academic databases, namely, MEDLINE, EMBASE, Web of Science, and J-STOR will be included—to allow for access to a diverse range of relevant studies conducted on South Africa between 2000 and 2015. Incidentally, this 15-year time horizon importantly marks an era of large-scale antiretroviral treatment (ART) coverage, classifiable as (i) ART introduction 2004–2007, (ii) expanded ART (2008–2010), and (iii) scaled-up ART (2011–present). Reasonably, this development was an appropriate setting for documenting implications on HIV-risk behavior and incidence patterns in key populations.

 See Additional file 1: Table S1 details the pilot search strategy considered to find relevant literature. In order to access additional relevant literature, the “cited by” reference searching feature (i.e., compliant with Web of Science) linking journal articles to other studies in which they have been cited will be considered. Search records will be documented for future references. We will also perform hand-searches on reference lists of studies initially discarded for thorough search of additional relevant primary studies to be included in this review. However, since referencing in literature reviews in general is almost inherently subject to citation bias, i.e., reviewing studies projecting only positive findings [24, 25], reference retrieval will largely remain a secondary search method.

(ii) Study selection

One reviewer will perform extensive title screening by searching and uploading all literature search results on a reference management software library, Mendeley Desktop, version 1.17.10 [26]. After which, all duplicates will be deleted from the library. Two independent reviewers will conduct extensive abstract screening to determine inclusion in a full-text review. Studies neither addressing our research questions nor meeting our inclusion criteria above will be excluded. Guiding the two independent reviewers in evaluating primary studies for full-text review will be the presumptive study selection procedure illustrated in Additional file 2: Figure S1. Full-text review will comprise of multiple rounds of scrutinizing studies for inclusion in the final round of selection depending on whether consensus is reached by the two reviewers that studies meet the inclusion criteria.

Any arising disagreements regarding inclusion will be resolved through consultation between review team members. The study selection outline will be summarized using the modified PRISMA flow-chart as indicated in Additional file 2: Figure S1.

(iii) Data extraction and appraisal

The final Mendeley database will be shared for abstract screening; at which stage, we will use two independent reviewers to extract data in parallel, from all relevant search engines. As a guide, we propose to use a systematized data extraction sheet (Additional file 3: Table S2). This sheet will capture bibliographic details, such as (a) study reference, (b) focus/aim of study, (c) study setting, and (d) HIV risk outcomes associated with migration. Additional file 3: Table S2 shows this in more detail.

#### Phases 2 and 3

(iv) Synthesis methods

We will present a qualitative and quantitative account of evidence from studies included in this review by way of a mixed-method systematic review sequential design. We will restrict the themes to the following presumptive outcomes: risky sexual practices and HIV incidence and frame them against other study and bio-demographic characteristics (Additional file 3: Table S2). The individual quantitative and qualitative syntheses will be conducted in parallel and then brought together in an overarching synthesis at a later stage.

##### Qualitative data

Qualitative data will be subjected to thematic synthesis, which largely involves the systematic coding of data and generating of descriptive and analytical themes. It is a particularly robust approach, which is essential given the aim to summarize textual evidence from primary studies according to emerging themes. First, data collected will be entered verbatim into a database and text will be coded according to its meaning and content. We will then generate descriptive themes, a process that basically involves translating concepts from each study and further categorizing codes according to similarities and differences. Lastly, we will develop analytical themes that go beyond mere factual descriptions or tables that for example, detail how many studies were assessed, the range of study sizes, and/or quality scores of each study as measured by a risk of bias tool [25]. Thus, this inductive process will sufficiently convey a deeper understanding of the key messages from existing data on risk behavior and HIV acquisition in South Africa.

##### Quantitative data

Guided by evidence obtained from the qualitative synthesis, we will aim to perform a meta-analysis (quantitative in nature). Specifically, we will identify studies reporting both univariate and multivariate statistics for the primary relationship examined here. Firstly, data are to be extracted from relevant primary studies via data management software, Distiller SR—which detects demographic and numerical values from text, amounting to a data quality management maneuver. We will explicitly restrict our data extraction to dichotomous data suitable for meta-analysis such as incident rate ratios and hazard ratios all at 95% confidence interval (CI). These estimates are desirable to quantify the effect of mobility on HIV risk acquisition particularly where results have positive association and significance, thus more sufficiently examining the unit of analysis. However, in instances where these statistics are unavailable, authors may be contacted. Notably, summary tables characterizing studies and results will be constructed for the meta-analysis of studies investigating the effects of migration on risk of HIV acquisition. Effect size data will be analyzed using a random-effects model, and a combined effect estimate will be computed through a meta-analysis. Heterogeneity of individual studies will be assessed using the combined effect size *I*^*2*^ statistic. The statistical analysis will be performed in STATA version 13.1 using pooled-effects estimates, and results will be presented on a forest plot.

##### Overarching synthesis

In this combined synthesis, authors will examine any emerging meaningful themes from the weight and pattern of the body of evidence based on results from the synthesized qualitative and quantitative data. This is particularly helpful to critically evaluate whether the totality of assembled evidence addressed the primary objective outlined in the introduction. Moreover, at this stage of the review, the authors will interpret and extrapolate study results so as to link significant findings with the key question in this review, effectively expressing the implications of study findings.

(v) Assessment of publication bias

For the purpose of ensuring that the most rigorous and appropriate measures and attempting to conduct a more valid review of empirical qualitative and mixed-method studies on mobility and HIV outcomes in South Africa, the Mixed Methods Appraisal Tool (MMAT) will be used to assess the methodological quality of studies and evidence of included studies. The tool’s validity and reliability have been well established; for one, it overcomes the inherent difficulty of appraising studies that use different methods by computing an overall quality score that varies by study design [19, 20], thus allowing one to obtain more in-depth answers to complex research questions often examined through studies of diverse designs. As such, we aim to use the most recent version of (MMAT), i.e., MMAT 2011 version.

To assess for publication bias in quantitative studies included in the systematic review and meta-analysis, we will conduct sensitivity analysis and plot results against the fit of a funnel plot. The basic assumption in the funnel plot is that studies with high precision will be plot near the average while studies with low precision will be spread evenly on either sides of the average, creating a somewhat funnel-shaped distribution. Essentially, deviation from this shape will suggest publication bias. Furthermore, the most recent guidelines of the preferred reporting items for systematic review and meta-analysis protocols (PRISMA-P) checklist was completed in validation of this study (Additional file 4).

## Discussion

Recent studies place important focus on key HIV vulnerable groups such as young women, migrants, and commercial sex workers (CSW). [15, 27, 28] to achieving effective combination HIV prevention strategies that target migrant populations. The intention of this study is to reveal evidence that link migrants and increased risk of HIV infections. The study aim is consistent with the UNAIDS current funding model and package of prevention strategies which involves clearly identifying main modes of HIV transmission, key affected populations, and core epidemiological trends for greater thrust on reducing new infections [4]. We intend to employ a conceptual framing supposing that migrant proximate and distal factors shape their risk to infection (including protective factors) to better provide the needed evidence base and justification for reliance on combination strategies and migrant-specific programs for HIV prevention in places.

The proposed systematic review study is set in a 15-year period (i.e., 2000–2015) in which the epidemic vastly evolved; findings may in one part be useful in confirming assumptions on the intersection of migration and sexual behavior outcomes and on the other challenging “old-age” beliefs on gender and HIV transmission such as the “male-infector model” [5]. The distinct association between HIV risk and CSW as well as truck driver lifestyle and occupations is a well-established finding in South Africa [1]. In addition, heterosexual exposure and behavior primarily account for most of the transmission in Sub-Saharan Africa [28]. As a result, in this review, we will exclude studies that report evidence on the following key populations: commercial sex workers (CSW), truck drivers, and men who have sex with men (MSM). Notably, only studies published in English will be included for the practical reason that most literature across the scientific establishment in South Africa and abroad are published in English language.

Reporting on key trends of the epidemic will provide requisite evidence to inform the focus of recent future work on migration and health and local HIV prevention programs sensitive to the socio-demographic dimensions of migrancy, typical sexual behaviors, and incidence burden. Results will be shared with the scientific community through publishing in a peer-reviewed journal and/or presenting at conferences.

## Additional files

Additional file 1: Sample search strategy in Web of Science. (DOCX 77 kb)

Additional file 2: PRISMA-P flow-chart of study selection procedure. (DOCX 48 kb)

Additional file 3: Data extraction summary table. (DOCX 14 kb)

Additional file 4: PRISMA-P (preferred reporting items for systematic review and meta-analysis protocols) 2015 checklist: recommended items to address in a systematic review protocol. (DOC 73 kb)

## Abbreviations

ART: Antiretroviral therapy
CSW: Commercial sex workers
HIV: Human immune virus
MSM: Men who have sex with men
PEOM: Population exposure outcome measurement
PRISMA: Preferred Reporting Items for Systematic Reviews and Meta-Analysis
PROSPERO: International prospective register for systematic reviews
SPIDER: Sample population of interest design evaluation research
UNAIDS: Joint United Nations Programme on HIV/AIDS

## References

[1] Weine SM, Kashuba AB (2012). Labor migration and HIV risk: a systematic review of the literature. AIDS Behav.

[2] Lurie MN, Williams BG. Migration and health in Southern Africa: 100 years and still circulating. Heal Psychol Behav Med [Internet]. 2014 Jan 1 [cited 2017 Sep 19];2(1):34–40. Available from: http://www.ncbi.nlm.nih.gov/pubmed/24653964. 10.1080/21642850.2013.866898 PMC3956074 24653964

[3] Cassels S, Jenness S, Khanna A. Conceptual framework and research methods for migration and HIV transmission dynamics. AIDS Behav [Internet]. 2014 [cited 2017 Jun 1]; Available from: http://link.springer.com/article/10.1007/s10461-013-0665-z. 10.1007/s10461-013-0665-z PMC4029933 24257897

[4] UNAIDS (2014). The gap report.

[5] Hunter M. Beyond the male-migrant: South Africa’s long history of health geography and the contemporary AIDS pandemic. Health Place [Internet]. 2010 [cited 2017 Sep 19]; Available from: http://www.sciencedirect.com/science/article/pii/S1353829209000793. 10.1016/j.healthplace.2009.08.003 19744874

[6] McGrath N, Eaton JW, Newell M-L, Hosegood V (2015). Migration, sexual behaviour, and HIV risk: a general population cohort in rural South Africa. Lancet HIV [Internet].

[7] Colebunders R, Kenyon C. Behaviour, not mobility, is a risk factor for HIV. Lancet HIV [Internet]. 2015 Jun [cited 2017 Aug 14];2(6):e223–4. Available from: http://www.thelancet.com/pdfs/journals/lanhiv/PIIS2352-3018(15)00057-0.pdf. 10.1016/S2352-3018(15)00057-0 26423190

[8] Coffee M, Lurie M, Garnett G. Modelling the impact of migration on the HIV epidemic in South Africa. Aids [Internet]. 2007 [cited 2017 May 31]; Available from: http://journals.lww.com/aidsonline/Abstract/2007/01300/Modelling_the_impact_of_migration_on_the_HIV.8.aspx. 10.1097/QAD.0b013e328011dac9 17255741

[9] Khan MR, Patnaik P, Brown L, Nagot N, Salouka S, Weir SS. Mobility and HIV-related sexual behavior in Burkina Faso. AIDS Behav [Internet]. 2008 Mar 30 [cited 2017 Sep 19];12(2):202–12. Available from: http://link.springer.com/10.1007/s10461-007-9314-8. 10.1007/s10461-007-9314-8 17968650

[10] Kishamawe C, Vissers DC, Urassa M, Isingo R, Mwaluko G, Borsboom GJ, et al. Mobility and HIV in Tanzanian couples: both mobile persons and their partners show increased risk. AIDS [Internet]. 2006 Feb [cited 2017 Sep 19];20(4):601–8. Available from: https://www.ncbi.nlm.nih.gov/pubmed/16470125. 10.1097/01.aids.0000210615.83330.b2 16470125

[11] Kwena ZA, Camlin CS, Shisanya CA, Mwanzo I, Bukusi EA. Short-term mobility and the risk of HIV infection among married couples in the fishing communities along Lake Victoria, Kenya. Tang J, editor. PLoS One [Internet]. 2013 Jan 15 [cited 2017 May 25];8(1):e54523. Available from: http://dx.plos.org/10.1371/journal.pone.0054523. 10.1371/journal.pone.0054523 PMC3545885 23336005

[12] Lagarde E, Schim van der Loeff M, Enel C, Holmgren B, Dray-Spira R, Pison G, et al. Mobility and the spread of human immunodeficiency virus into rural areas of West Africa. Int J Epidemiol [Internet]. 2003 Oct 1 [cited 2017 Sep 19];32(5):744–52. Available from: https://academic.oup.com/ije/article-lookup/doi/10.1093/ije/dyg111. 10.1093/ije/dyg111 14559743

[13] Camlin C, Hosegood V, Newell M, McGrath N. Gender, migration and HIV in rural KwaZulu-Natal, South Africa. PLoS One [Internet]. 2010 [cited 2017 Feb 8]; Available from: http://journals.plos.org/plosone/article?id=10.1371/journal.pone.0011539. 10.1371/journal.pone.0011539 PMC2902532 20634965

[14] Voeten H, Vissers D, Gregson S. Strong association between in-migration and HIV prevalence in urban sub-Saharan Africa. Sex Transm [Internet]. 2010 [cited 2017 Jun 1]; Available from: https://www.ncbi.nlm.nih.gov/pmc/articles/PMC3514976/. 10.1097/OLQ.0b013e3181c3f2d0 PMC3514976 19959971

[15] Dobra A, Bärnighausen T, Vandormael A, Tanser F. Space-time migration patterns and risk of HIV acquisition in rural South Africa. AIDS [Internet]. 2017 Jan 2 [cited 2017 Jun 1];31(1):137–45. Available from: http://www.ncbi.nlm.nih.gov/pubmed/27755099. 10.1097/QAD.0000000000001292 PMC5131684 27755099

[16] Collinson MA. Striving against adversity: the dynamics of migration, health and poverty in rural South Africa. Glob Health Action [Internet]. 2010 Dec 3 [cited 2017 Aug 16];3(1):5080. Available from: https://www.tandfonline.com/doi/full/10.3402/gha.v3i0.5080. 10.3402/gha.v3i0.5080 PMC2882287 20531981

[17] Mah TL, Halperin DT (2010). Concurrent sexual partnerships and the HIV epidemics in Africa: evidence to move forward. AIDS Behav.

[18] Saggurti N, Nair S, Malviya A, Decker MR, Silverman JG, Raj A. Male migration/mobility and HIV among married couples: cross-sectional analysis of nationally representative data from India. AIDS Behav [Internet]. 2012 Aug 3 [cited 2017 May 25];16(6):1649–58. Available from: http://journals.plos.org/plosone/article?id=10.1371/journal.pone.0043222. 10.1007/s10461-011-0022-z 21811841

[19] Pluye P, Robert E, Cargo M, Bartlett G, O’Cathain A. Proposal: a mixed methods appraisal tool for systematic mixed studies reviews. Montréal McGill Univ [Internet]. 2011 [cited 2017 Sep 19]; Available from: http://mixedmethodsappraisaltoolpublic.pbworks.com.

[20] Pace R, Pluye P, Bartlett G, Macaulay AC, Salsberg J, Jagosh J, et al. Testing the reliability and efficiency of the pilot Mixed Methods Appraisal Tool (MMAT) for systematic mixed studies review. [cited 2017 Sep 19]; Available from: https://www.ncbi.nlm.nih.gov/pubmed/21835406. 10.1016/j.ijnurstu.2011.07.002 21835406

[21] Gough D, Oliver S, Thomas J. An introduction to systematic reviews. 2nd edition. London: Sage Publications Ltd, 304 pages. ISBN: 9781849201810. 2017 Mar [cited 2017 Sep 20];48(3):369–83. Available from: https://eppi.ioe.ac.uk/cms/Resources/tabid/88/Default.aspx?tabid=273.

[22] Arksey H, O’Malley L. Scoping studies: towards a methodological framework. Int J Soc Res Methodol [Internet]. 2005 Feb [cited 2017 Sep 20];8(1):19–32. Available from: http://www.tandfonline.com/doi/abs/10.1080/1364557032000119616.

[23] Cooke A, Smith D, Booth A. Beyond PICO. Qual Health Res [Internet]. 2012 Oct 24 [cited 2017 Dec 8];22(10):1435–43. Available from: http://journals.sagepub.com/doi/10.1177/1049732312452938. 10.1177/1049732312452938 22829486

[24] Bettany-Saltikov J. Learning how to undertake a systematic review: part 2. Nurs Stand [Internet]. 2010 Aug 25 [cited 2017 Dec 8];24(51):47–56. Available from: http://rcnpublishing.com/doi/abs/10.7748/ns2010.08.24.51.47.c7943. 10.7748/ns2010.08.24.51.47.c7943 20860325

[25] Krumpal I. Determinants of social desirability bias in sensitive surveys: a literature review. Qual Quant [Internet]. 2013 Jun 19 [cited 2017 Sep 20];47(4):2025–47. Available from: http://link.springer.com/10.1007/s11135-011-9640-9.

[26] Mendeley. Getting started with Mendeley*.* Mendeley Desktop*.* London: 2009 Mendeley Ltd. Retrieved from http://www.mendeley.com/.

[27] Wamai RG, Morris BJ, Bailis SA, Sokal D, Klausner JD, Appleton R, et al. Male circumcision for HIV prevention: current evidence and implementation in sub-Saharan Africa. J Int AIDS Soc [Internet]. 2011 Oct 20 [cited 2017 Sep 26];14(1):49. Available from: http://www.ncbi.nlm.nih.gov/pubmed/22014096. 10.1186/1758-2652-14-49 PMC3207867 22014096

[28] Townsend L, Giorgio M, Zembe Y, Cheyip M. HIV prevalence and risk behaviours among foreign migrant women residing in Cape Town, South Africa. AIDS Behav [Internet]. 2014 [cited 2017 Feb 8]; Available from: http://link.springer.com/article/10.1007/s10461-014-0784-1. 10.1007/s10461-014-0784-1 PMC5778353 24781639

