# Supplementary material for: Mobility and increased risk of HIV acquisition in South Africa: a mixed-method systematic review protocol
Source: Syst Rev. 2018 Feb 27;7:37. doi: 10.1186/s13643-018-0703-z (PMC6389209; doi:10.1186/s13643-018-0703-z)
Supplement: Supplementary file 1 — Sample search strategy in Web of Science. (DOCX 77 kb) [file 13643_2018_703_MOESM1_ESM.docx]

**Additional File 1: Sample search strategy in Web of Science**

| Sample | (“migrants” [Title] OR “male migrants” [Title] OR “female migrants” [Title] OR “mobile people” [Mesh Major Topic] |
| --- | --- |
| Phenomenon of interest: migration | (“migration” [MeSH Major Topic]) OR (“Migrat*”  [Title]) OR (“rural to urban migration [MeSH Major Topic]) OR (“temporary migration” [MeSH Major Topic]) OR (“circular migration” [Title]) OR (“oscillating migration” [MeSH]) OR (“mobility” [Title]) OR (“outmigration” [Mesh Major Topic]) |
| Evaluation | (“multiple sexual partners” [MeSH Major Topic]) OR (“HIV risk” [Title]) OR (HIV*[Title] OR “transmission risk”) [Title] OR (“AIDS risky behavior” [MeSH Major Topic]) OR (“concurrent sexual partners” [MeSH Major Topic]) OR (“transactional sex” [MeSH Major Topic]) OR (“casual sex” [MeSH Major Topic]) OR (“unprotected sex” [MeSH Major Topic]) OR (“low HIV knowledge” [Mesh Major Topic]) OR (“low perceived risk of HIV infection” [Mesh Major Topic]) OR (“HIV incidence*”[Title]) OR (“HIV infection*”[Title]”) OR (“HIV transmission*”[Title]) OR (“risk of HIV acquisition” [MeSH Major Topic]) OR (“new infections” [Title]) OR (“sero-incidence” [MeSH Major Topic]) (heterosexual transmission [MeSH Major Topic] |

Table S1

Note: This preliminary search strategy is in accordance with some elements of the SPIDER criteria
