# Supplementary material for: Mobility and increased risk of HIV acquisition in South Africa: a mixed-method systematic review protocol
Source: Syst Rev. 2018 Feb 27;7:37. doi: 10.1186/s13643-018-0703-z (PMC6389209; doi:10.1186/s13643-018-0703-z)
Supplement: Supplementary file 2 — PRISMA-P flow-chart of study selection procedure. (DOCX 48 kb) [file 13643_2018_703_MOESM2_ESM.docx]

**Additional File 2: PRISMA-P flow-chart of study selection procedure**

Studies obtained through hand searching

Initial results through database searching

## Identification

Duplicate records removed

## Screening

Records screened using titles & abstracts

Records excluded

Full-text articles assessed for eligibility

Full-text articles excluded

## Eligibility

Studies included in systematic review

Qualitative studies included

## Included

Mixed method studies included

Quantitative studies included

Studies excluded

Studies included for meta-analysis

Fig S1
