# Supplementary material for: Mobility and increased risk of HIV acquisition in South Africa: a mixed-method systematic review protocol
Source: Syst Rev. 2018 Feb 27;7:37. doi: 10.1186/s13643-018-0703-z (PMC6389209; doi:10.1186/s13643-018-0703-z)
Supplement: Supplementary file 3 — Data extraction summary table. (DOCX 14 kb) [file 13643_2018_703_MOESM3_ESM.docx]

| **Reference/Author and date** | **Study title** | **Study location** | **Socio-demographic profile** | **Focus/Aim of study** | **Methodology** | **Sample size** | **Marital status** | **Migration dimension** | **Sexual risk behavior** | **HIV infection (incidence)** | **Statistical results** |
| --- | --- | --- | --- | --- | --- | --- | --- | --- | --- | --- | --- |
|  |  |  |  |  |  |  |  |  |  |  |  |

**Additional File 3: Data extraction summary table**

Table S2

Notes: Data from included studies will be extracted onto the presumptive format above. Reference details study design, bio-demographic profile of participants, focus of study, sample size, measure of migration, sexual risk outcome and HIV incidence statistics will be displayed will be displayed.
