# Supplementary material for: Mobility and increased risk of HIV acquisition in South Africa: a mixed-method systematic review protocol
Source: Syst Rev. 2018 Feb 27;7:37. doi: 10.1186/s13643-018-0703-z (PMC6389209; doi:10.1186/s13643-018-0703-z)
Supplement: Supplementary file 4 — PRISMA-P (preferred reporting items for systematic review and meta-analysis protocols) 2015 checklist: recommended items to address in a systematic review protocol. (DOC 73 kb) [file 13643_2018_703_MOESM4_ESM.doc]

| **Section and topic** | **Item No** | **Checklist item** | **Reported on page No** |
| --- | --- | --- | --- |
| **Title** | | |  |
| Title | 1 | Identify the report as a systematic review, meta-analysis, or both. | Page 1 |
| Identification | 1a | Identify the report as a protocol of a systematic review | Page1 |
| Update | 1b |  | N/A |
| Registration | 2 | If registered, provide the name of the registry (such as PROSPERO) and registration number | Page 3 |
| Authors |  |  |  |
| Contact: | 3a | Provide name, institutional affiliation, e-mail address of all protocol authors; provide physical mailing address of corresponding author | Page 2 |
| Contributions | 3b | Describe contributions of protocol authors and identify the guarantor of the review | Page 18 |
| Amendments | 4 | If the protocol represents an amendment of a previously completed or published protocol, identify as such and list  changes; otherwise, state plan for documenting important protocol amendments | N/A |
| Support: |  |  |  |
| Sources | 5a | Indicate sources of financial or other support for the review | Page 18 |
| Sponsor | 5b | Provide name for the review funder and/or sponsor | Page 18 |
| Role of sponsor or funder | 5c | Describe roles of funder(s), sponsor(s), and/or institution(s), if any, in developing the protocol | N/A |
| **Introduction** | | |  |
| Rationale | 6 | Describe the rationale for the review in the context of what is already known. | Pages 3, 4, 5 |
| Objectives | 7 | Provide an explicit statement of questions being addressed with reference to population, exposure, outcomes, and measurement (PEOM) and sample, population of interest, design, evaluation, research study (SPIDER). | Page 6, 7, 8 & 9 |
| **Methods** | | |  |
| Protocol and registration | 8 | Indicate if a review protocol exists, if and where it can be accessed (e.g., Web address), and, if available, provide registration information including registration number. | Page 5 |
| Eligibility criteria | 9 | Specify study characteristics (e.g., PICOS, length of follow-up) and report characteristics (e.g., years considered, language, publication status) used as criteria for eligibility, giving rationale. | Pages 10 & 11 |
| Information sources | 10 | Describe all information sources (e.g., databases with dates of coverage, contact with study authors to identify additional studies) in the search and date last searched. | Page 10 |
| Search strategy | 11 | Present full electronic search strategy for at least one database, including any limits used, such that it could be repeated. | Pages 7 & 9 |
| Study records: |  |  |  |
| Data management | 12a | Describe the mechanism(s) that will be used to manage records and data throughout the review | Page 11 |
| Selection process | 12b | State the process for selecting studies (i.e., screening, eligibility, included in systematic review, and, if applicable, included in the meta-analysis). | Page 12 |
| Data collection process | 12c | Describe method of data extraction from reports (e.g., piloted forms, independently, in duplicate) and any processes for obtaining and confirming data from investigators. | Page 12 |
| Data items | 13 | List and define all variables for which data were sought (e.g., PICOS, funding sources) and any assumptions and simplifications made. | Page 12 |
| Outcomes and prioritization | 14 | List and define all outcomes for which data will be sought, including prioritization of main and additional outcomes,  with rationale | Page 12 |
| Risk of bias in individual studies | 15 | Describe methods used for assessing risk of bias of individual studies (including specification of whether this was done at the study or outcome level), and how this information is to be used in any data synthesis. | Page 14 & 15 |
| Data synthesis | 16a | Describe criteria under which study data will be quantitatively synthesised | Page 13 & 14 |
|  | 16b | State the principal summary measures (e.g., risk ratio, difference in means). | Page 13 |
|  | 16c | Describe the methods of handling data and combining results of studies, if done, including measures of consistency (e.g., I^2^) for each meta-analysis. | Page 14 |
|  | 16d | If quantitative synthesis is not appropriate, describe the type of summary planned | Pages 13 &14 |
| Meta-bias(es) | 17 | Specify any planned assessment of meta-bias(es) (such as publication bias across studies, selective reporting within  studies) | Pages 14 |
| Confidence in cumulative evidence | 18 | Describe how the strength of the body of evidence will be assessed (such as GRADE) | Pages 14 |
| Additional analyses | 19 | Describe methods of additional analyses (e.g., sensitivity or subgroup analyses, meta-regression), if done, indicating which were pre-specified | Page 14 |
| **Discussion** |  |  |  |

| **DISCUSSION** | | |  |
| --- | --- | --- | --- |
| Summary of evidence | 24 | Summarize the main findings including the strength of evidence for each main outcome; consider their relevance to key groups (e.g., healthcare providers, users, and policy makers). | Page 15-16 |
| Limitations | 25 | Discuss limitations at study and outcome level (e.g., risk of bias), and at review-level (e.g., incomplete retrieval of identified research, reporting bias). | Page 16 |
| Conclusions | 26 | Provide a general interpretation of the results in the context of other evidence, and implications for future research. | Page 16 |
| **FUNDING** | | |  |
| Funding | 27 | Describe sources of funding for the systematic review and other support (e.g., supply of data); role of funders for the systematic review. | Page 18 |

*From:*  Shamseer L, Moher D, Clarke M, Ghersi D, Liberati A, Petticrew M, Shekelle P, Stewart LA. Preferred reporting items for systematic review and meta-analysis protocols (PRISMA-P) 2015: elaboration and explanation. Bmj. 2015 Jan 2;349:g7647.

For more information, visit: **www.prisma-statement.org**.

Page 2 of 2
